# Identification of four Drosophila Toll-related proteins as ligands for the PTP69D receptor tyrosine phosphatase

**DOI:** 10.17912/micropub.biology.000159

**Published:** 2019-09-03

**Authors:** Namrata Bali, Hyung-Kook Lee, Kai Zinn

**Affiliations:** 1 Division of Biology and Biological Engineering, California Institute of Technology, 1200 E. California Blvd., Pasadena, CA, 91125, USA.

**Figure 1. Toll-2, Toll-6, Toll-7 and Toll-8 bind to PTP69D:**
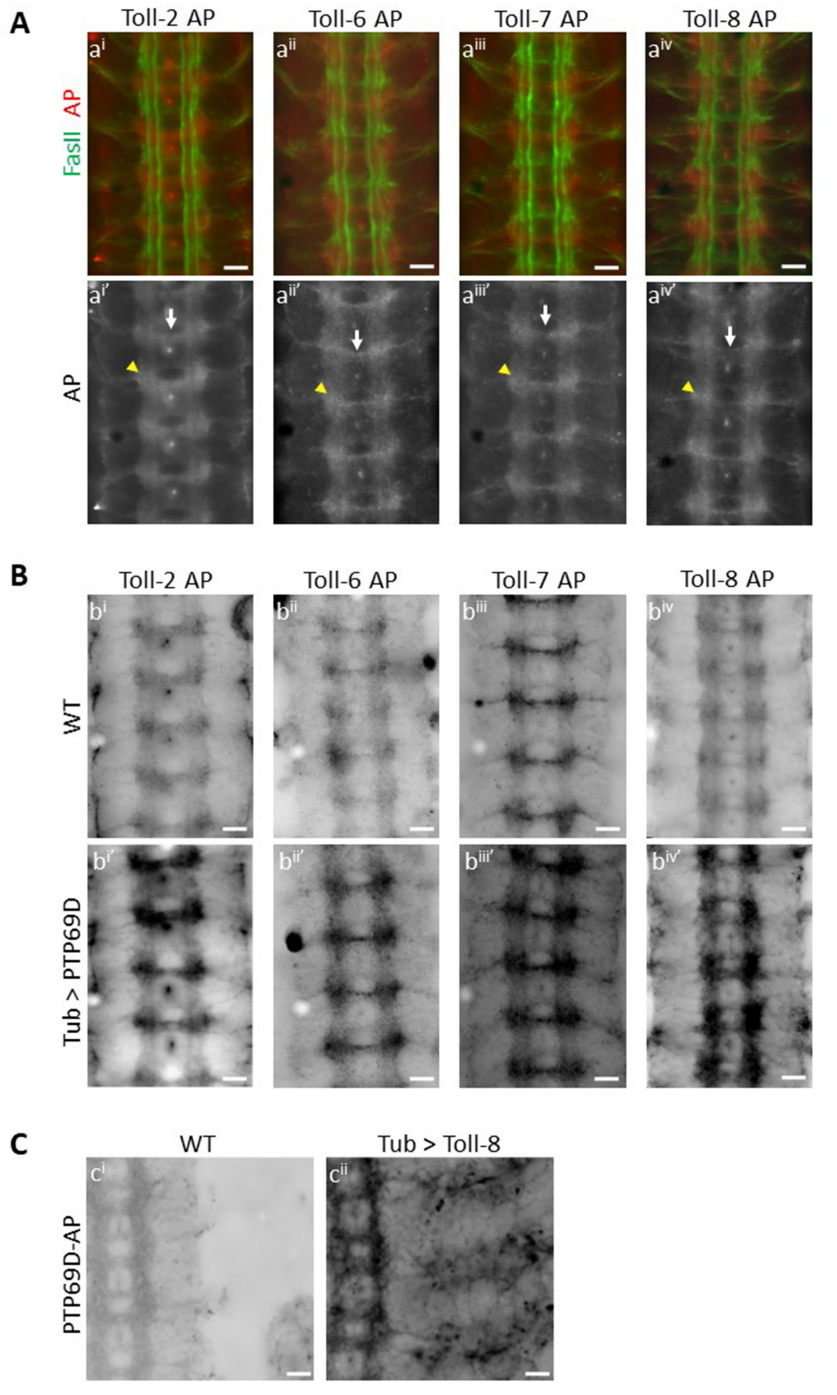
(A) Binding patterns of Toll-2 AP, Toll-6 AP, Toll-7 AP and Toll-8 AP fusion proteins in wild type embryos. a^i^-a^iv^ show double staining for FasII (green) and Toll-x AP (red). In a^i’^-a^iv’^ only the AP signal is shown, in white. All four fusion proteins show a similar binding pattern in the CNS with AP staining seen in longitudinal CNS axons, junctions between the longitudinal and the commissural tracts (yellow arrowheads), and in the posterior commissure (white arrows). Weak binding is also seen in the anterior commissure and in motor axons leaving the CNS. (B) Toll-2, Toll-6, Toll-7 and Toll-8 bind to overexpressed PTP69D. Wild type embryos (b^i^ – b^iv^) and embryos with overexpressed PTP69D (b^i’^ – b^iv’^) were incubated with Toll-2 AP, Toll-6 AP, Toll-7 AP and Toll-8 AP fusion proteins. Here only the AP signal is shown, with black and white reversed relative to (a), so that AP staining is black. Embryos with ectopic PTP69D show increased binding of AP fusion proteins compared to wild type embryos. (C) PTP69D binds to overexpressed Toll-8. Wild type embryos (c^i^) and embryos with ectopic Toll-8 (c^ii^) were incubated with PTP69D-AP fusion protein. Embryos with overexpressed Toll-8 show increased binding by PTP69D-AP (black) both in the CNS and in the periphery. Scale bars, 10µm.

## Description

The nine Toll-related receptors in *Drosophila* (Toll-1 – Toll-9) (Valanne *et al.* 2011) mediate a range of functions, ranging from embryonic development and immunity (Valanne *et al.* 2011) to larval locomotion, motor axon targeting and neuronal survival (McIlroy *et al.* 2013). Some members of the Toll family in *Drosophila* have been shown to bind to members of the Spaetzle family (Valanne *et al.* 2011; McIlroy *et al.* 2013; Ballard *et al.* 2014). Toll-6 and Toll-7 bind to Spz2 and Spz5 in a promiscuous manner (McIlroy *et al.* 2013). Toll-8 (also known as Tollo) has been shown to bind to Spz3 (Ballard *et al.* 2014).

Our group has conducted several screens to identify ligands for *Drosophila* receptor-like protein tyrosine phosphatases (RPTPs). A deficiency screen identified Syndecan as a ligand for Lar (Fox and Zinn 2005), and a gain-of-function screen identified Stranded at second (Sas) as a ligand for PTP10D (Lee *et al.* 2013). Here we show that members of the Toll family are ligands for PTP69D, an RPTP expressed exclusively on CNS axons in the embryo.

Extracellular domains (ECD) of cell surface and secreted proteins can be used to stain live embryos, and the observed binding patterns may represent the expression patterns of ligand(s) for these ECDs (Fox and Zinn 2005; Lee *et al.* 2013; Ozkan *et al.* 2013). Here, we used ECDs of Toll proteins fused to pentameric Alkaline Phosphatase (AP) to create AP fusion proteins (Ozkan *et al.* 2013). Live-dissected late stage 16 *Drosophila* embryos were incubated with these AP fusion proteins, using methods described in (Bali *et al.* 2016), to reveal *in vivo* binding patterns of Toll proteins in the *Drosophila* CNS. Wild-type embryos were incubated with Toll-2 (also known as 18w) AP, Toll-6 AP, Toll-7 AP and Toll-8 AP separately and immunostained for AP and FasII. Surprisingly, we observed a similar binding pattern for the four Toll proteins, suggesting common binding partners. All four Toll proteins showed binding to longitudinal CNS axons (A), and maximum staining intensity was observed at the junctions between the longitudinal and the commissural tracts (A, a^i’^ – a^iv’^, yellow arrowheads). These regions are where many synaptic connections between neurons projecting in the longitudinal and commissural tracts will later form. Binding of Toll proteins was also seen to a bundle of axons in the posterior commissure (A, a^i’^ – a^iv’^, white arrows). The anterior commissure was weakly labeled. Weak binding was also seen to motor axons as they leave the CNS. No binding was seen to muscles for any of the Toll proteins examined (data not shown).

We had identified Toll-8 as a putative ligand for PTP69D in the gain-of-function embryo binding screen. In this screen, RPTP-AP proteins were used to stain embryos from crosses of a collection of ~300 lines with UAS-containing P elements upstream of cell surface protein genes to a pancellular GAL4 driver line (Lee *et al.* 2013). Since Toll-2, Toll-6 and Toll-7 showed a similar binding pattern to Toll-8 in wild-type embryos, we sought to examine whether they also bind to PTP69D. PTP69D was ectopically expressed in embryos by crossing tubulin-GAL4 to a line with an insertion of a UAS-containing P element in the 5’ end of the PTP69D gene. This conferred overexpression of PTP69D, especially in the CNS. Both wild-type embryos and embryos with ectopic PTP69D expression were incubated with Toll2-AP, Toll6-AP, Toll7-AP and Toll8-AP in separate experiments. In each case, we saw significantly increased binding of individual AP fusion proteins to the ectopically expressed PTP69D (B, compare b^i^ to b^i’^, b^ii^ to b^ii’^, b^iii^ to b^iii’^ and b^iv^ to b^iv’^). This shows that all four Toll proteins examined are able to bind to ectopically expressed PTP69D. Interestingly, although PTP69D expression was driven using a pancellular driver, we observed increased staining only on CNS axons. This suggests that the Toll proteins might be able to bind to PTP69D only when a cofactor expressed in the CNS is present. Alternatively (or in addition), PTP69D might only be able to localize to the cell surface on CNS axons.

Here we also show Toll-8 binding to PTP69D in the reverse orientation, as in the gain-of-function screen. We ectopically expressed Toll-8 using tubulin-GAL4 and a line with a UAS-containing P element insertion upstream of the Toll-8 gene. We incubated both wild-type embryos and embryos with ectopic expression of Toll-8 with PTP69D-AP fusion protein and saw greatly increased binding of 69D-AP fusion protein to ectopically expressed Toll-8, both in the CNS and in the periphery. In the CNS, staining is observed only on axons and not on cell bodies, suggesting that Toll-8 localizes to axons, as does PTP69D. Thus Toll-8 and PTP69D bind to each other *in vivo* when either is over-expressed. Taken together, our results show that we have identified four Toll proteins that are likely to be novel ligands for PTP69D, either individually or as part of a larger complex.

## Methods

AP fusion protein generation: Expression plasmids containing ECDs of Toll2, Toll6, Toll7 and Toll8 with AP domains were obtained from (Ozkan *et al.* 2013). Briefly, S2 cells were transiently transfected were individual expression plasmids using Effectene transfection reagent (Qiagen). Expression of AP fusion proteins was induced by addition of 1mM copper sulfate. AP fusion protein containing cell supernatant (Sup) was collected 3 days after induction. 1x Sup was concentrated five-fold using Amicon Ultra Centrifugal Filters and stored at 4°C.

Live-dissection of Drosophila embryos and staining: For detailed description of dissection and staining protocol used, see (Lee *et al.* 2009; Bali *et al.* 2016). Briefly, after live dissections of stage 16 embryos, they were incubated with 5x AP fusion proteins for two hours, followed by fixation. Immunostaining was performed against AP (Rabbit anti-AP, AbD Serotec) and FasII (Mouse anti-1D4, DSHB). Images were captured using Olympus Fluorescence microscope with the same acquisition parameters used for all images. Each experiment consisted of 6-10 embryos of each genotype on the same slide per fusion protein. Ectopic binding was seen in ~70% – 90% embryos. Experiments were repeated at least 3 times for each fusion protein

## Reagents

y^1^ w^1^ (FlyBase ID FBst0001495)

tubulin-GAL4 (FlyBase ID FBal0181584)

P{EPgy2}PTP69D[EY04186] (UAS-PTP69D) (FlyBase ID FBti0027297)

P(XP)d01565 (UAS-toll8) (FlyBase ID FBst1009303)

## References

[R1] Bali N, Lee HK, Zinn K (2016). Live Staining of Drosophila Embryos with RPTP Fusion Proteins to Detect and Characterize Expression of Cell-Surface RPTP Ligands.. Methods Mol Biol.

[R2] Ballard SL, Miller DL, Ganetzky B (2014). Retrograde neurotrophin signaling through Tollo regulates synaptic growth in Drosophila.. J Cell Biol.

[R3] Fox AN, Zinn K (2005). The heparan sulfate proteoglycan syndecan is an in vivo ligand for the Drosophila LAR receptor tyrosine phosphatase.. Curr Biol.

[R4] Lee HK, Cording A, Vielmetter J, Zinn K (2013). Interactions between a receptor tyrosine phosphatase and a cell surface ligand regulate axon guidance and glial-neuronal communication.. Neuron.

[R5] Lee HK, Wright AP, Zinn K (2009). Live dissection of Drosophila embryos: streamlined methods for screening mutant collections by antibody staining.. J Vis Exp.

[R6] McIlroy G, Foldi I, Aurikko J, Wentzell JS, Lim MA, Fenton JC, Gay NJ, Hidalgo A (2013). Toll-6 and Toll-7 function as neurotrophin receptors in the Drosophila melanogaster CNS.. Nat Neurosci.

[R7] Özkan E, Carrillo RA, Eastman CL, Weiszmann R, Waghray D, Johnson KG, Zinn K, Celniker SE, Garcia KC (2013). An extracellular interactome of immunoglobulin and LRR proteins reveals receptor-ligand networks.. Cell.

[R8] Valanne S, Wang JH, Rämet M (2011). The Drosophila Toll signaling pathway.. J Immunol.

